# Supplementary material for: Type-I CdSe@CdS@ZnS Heterostructured Nanocrystals with Long Fluorescence Lifetime
Source: Materials (Basel). 2023 Nov 1;16(21):7007. doi: 10.3390/ma16217007 (PMC10648168; doi:10.3390/ma16217007)
Supplement: Supplementary file 1 [file materials-16-07007-s001.zip › materials-2672494-supplementary.pdf]

catalysts

Supporting Information

Experimental Section

Synthesis of CdSe@CdS@ZnSe

First, to obtain the precursor, we weighed 1.2 mmol of selenium powder and added it to a 5 mL test flask with 2 mL of tri-n-octylphosphine (TOP). The mixture was then subjected to pre-ultrasonic dispersion and dissolution. Then, we added 0.6 mmol of zinc acetate to a three-necked flask containing 2 mL of oleic acid (OAc) and 6 mL of 1-octadecene (ODE). The flask was then mounted on a heating magnetic stirrer. The mixture was degassed at 120 °C for 30 min and then heated at 250 °C for 1 h under a nitrogen atmosphere, to ensure complete dissolution of the solid. After that, the solution was cooled to 70 °C, and 1 mL of CdSe@CdS chloroform solution was quickly injected into the flask and degassed for 30 min, followed by heating at 250 °C under a nitrogen atmosphere. The precursor prepared before was slowly injected to the above solution at a rate of 0.1 mL/min. It was reacted another 40 min after injection. The solution was cooled to room temperature and centrifuged 1-2 times with 5 mL hexane and 15 mL ethanol. Finally dispersed in 5 mL $CHCl_3$ for further characterizations.

Photoluminescence (PL) measurements

The sample solutions were diluted with hexane, to reach an optical density of ~0.1–0.2 at the excitation wavelength. The steady-state PL spectra were collected with a spectrofluorometer (Hitachi F-7000), and the sample was excited by Xe lamp coupled monochromator with an excitation wavelength of 400 nm. For photoluminescence quantum yield (PLQY) measurements, the spectra were collected using a spectrofluorimeter (Edinburgh Instruments FLS1000), by exciting the sample with a monochromated xenon lamp source. The central wavelength was set to λ_{ex} = 400 nm for the samples. The exciting light was coupled into an optical fiber connected to an integrating sphere where a quartz cuvette containing the sample was placed. The system consists of a spectrometer and a PMT detector. The excitation light is coupled to the optical fiber connected to the integrating sphere, and a quartz cuvette containing the sample is placed in the integrating sphere. Then, the emitted light is collected from the sphere and the second fiber is coupled to the detection system for detection.

For each sample, four measurements were performed: (1) the sample emission (SEM) that collects the photons emitted by the sample; (2) the blank emission (BEM), which is a measurement performed with the cuvette containing only the solvent (blank) in the same spectral range used for the SEM measurement; (3) the sample excitation (SEX), which records the photons at the pumping wavelength that are not absorbed by the sample; (4) the blank excitation (BEX), which records the photons at the pumping wavelength going through the blank[43]. The PLQY was then calculated as

$$PLQY(\%) = \frac{SEM-BEM}{BEX-SEX} \qquad (1)$$

Any reabsorption correction factor was neglected in our calculation of the PLQY, since the solutions we prepared were diluted to the point that reabsorption of the PL could be neglected. Time-resolved PL measurements were measured using a spectrofluorimeter (Edinburgh Instruments FLS1000).

Fitting formulas

The attenuation curve of the prepared samples has a good fitting with biexponential functions, and the corresponding attenuation equation is[44]:

$$I = A_1 \exp(-\frac{t}{\tau_1}) + A_2 \exp(-\frac{t}{\tau_2}) \qquad (2)$$

Where I represents the relative intensity of fluorescence, τ_1 and τ_2 represent the two components of fluorescence lifetime, A_1 and A_2 represent constants. The average fluorescence lifetime was estimated using the following formula:

$$\tau_{\mathrm{Avg}} = \frac{A_1 {\tau_1}^2 + A_2 {\tau_2}^2}{A_1 \tau_1 + A_2 \tau_2} \qquad (3)$$

Figures and Tables

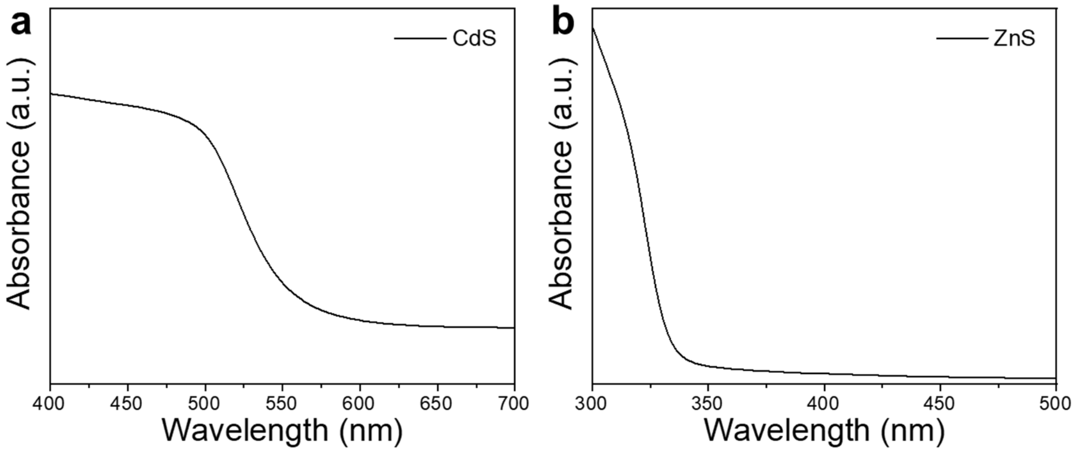

Figure S1. UV-vis absorbance spectra of (a) CdS and (b) ZnS.

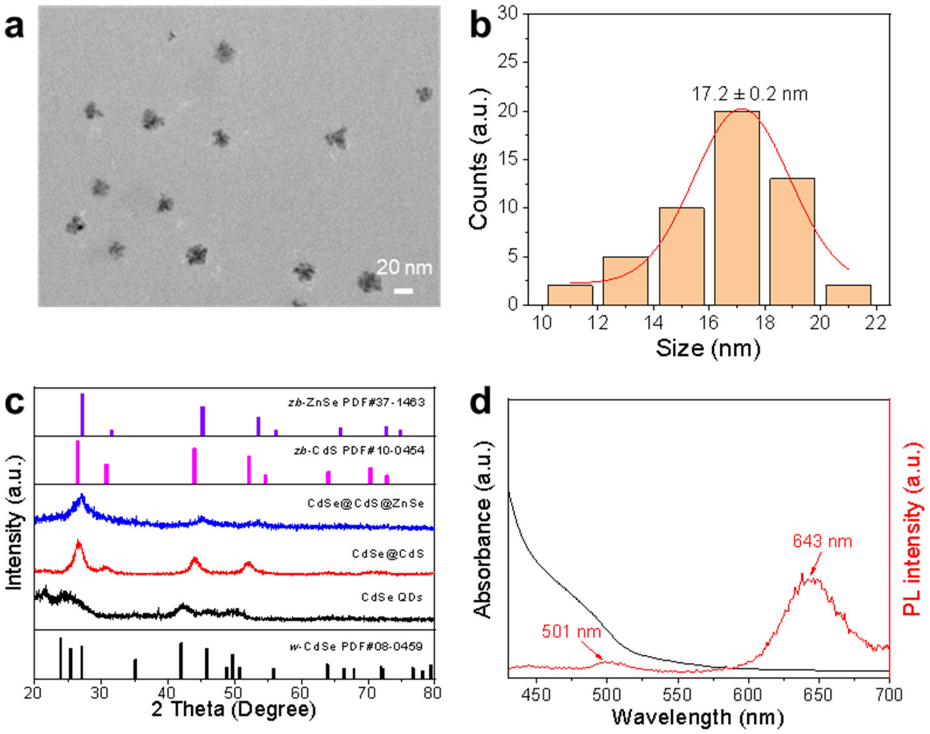

Figure S2. (a) Representative TEM images CdSe@CdS@ZnSe; (b) Size distribution diagrams of CdSe@CdS@ZnSe; (c) XRD patterns of CdSe QDs, CdSe@CdS and CdSe@CdS@ZnSe; (d) UV-vis absorbance spectra and photoluminescence (PL) spectra of CdSe@CdS@ZnSe.

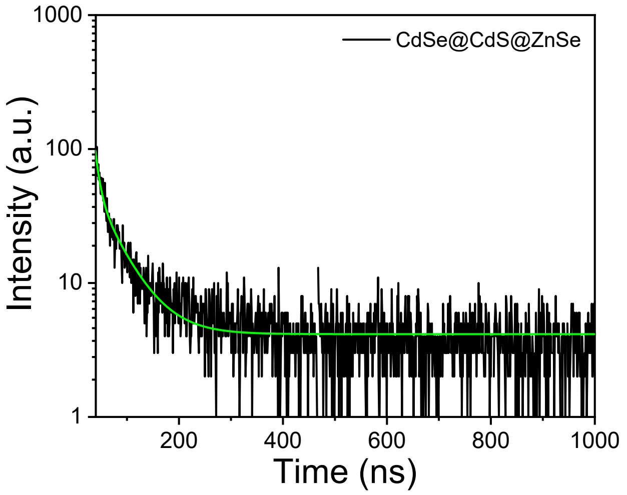

Figure S3. Time-resolved PL at the main peak for CdSe@CdS@ZnSe. Best fit in green line using biexponential.

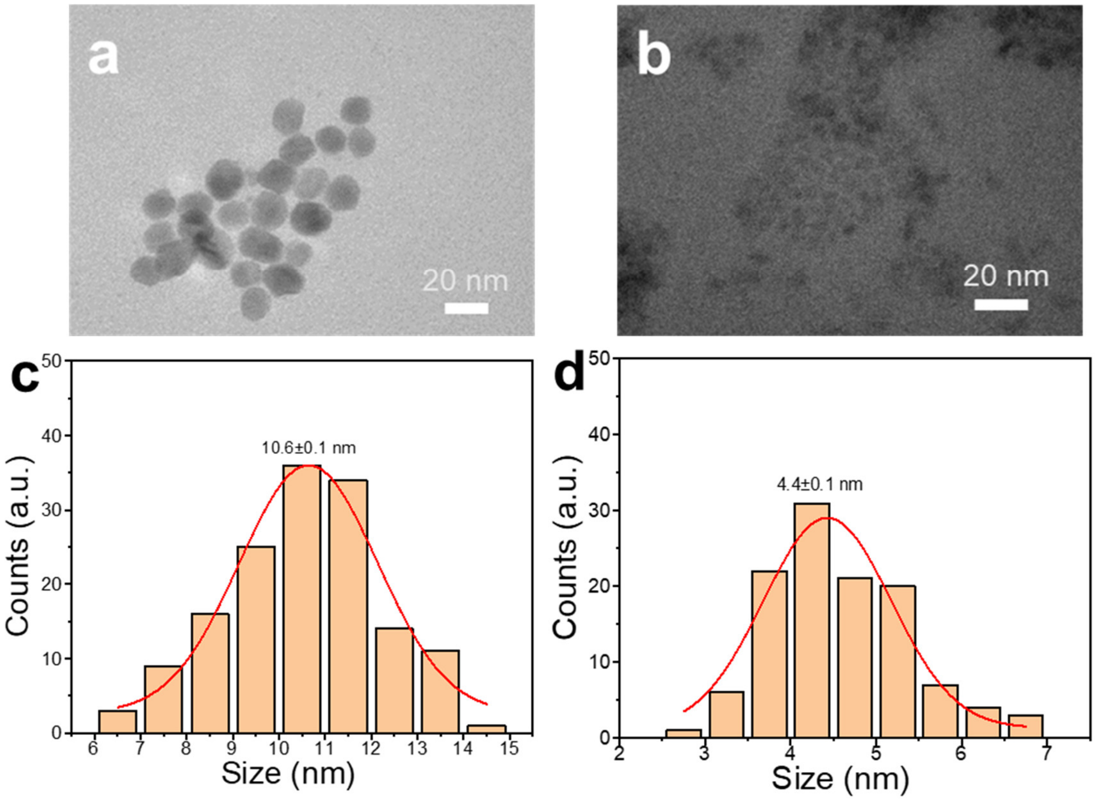

Figure S4. Representative TEM images of (a) CdS; (b) ZnS; Size distribution diagrams of (c) CdS; (d) ZnS.

Table S1. X, E_g, E_{CB} and E_{VB} of CdSe, CdS and ZnS.

Samples	X (eV)	E_g (eV)	E_{CB} (eV)	E_{VB} (eV)
CdSe	5.05	1.94	-0.42	1.52
CdS	5.19	2.12	-0.37	1.75
ZnS	5.26	3.62	-1.05	2.57

Table S2. Time-resolved PL kinetics of CdSe QDs, CdSe@CdS and CdSe@CdS@ZnS upon the excitation of 400 nm. Fitting procedures are described by biexponential function analysis.

Samples	Fitting parameters					
	**τ_1 (ns)**	**A_1**	**τ_2 (ns)**	**A_2**	τ_{Avg} (ns)	PLQY
CdSe QDs	85.99	1084	29.09	11624	41.4	15.89%
CdSe@CdS	92.88	1541	36.53	5898	59.0	6.32%
CdSe@CdS@ZnS	219.24	332.6	52.25	4977	88.8	17.05%

Table S3. Comparison of the reported photoluminescence lifetimes of different core-shell materials.

**Samples**	**PL Lifetime**	**Reference**
CdSe@CdS@ZnS	**88.8 ns**	**This Paper**
CdSe/CdS/ZnS	53.0 ns	[1]
CdSe/CdS/CdZnS/ZnS	76.1 ns	[2]
CdSe/CdS	29.2 ns	[3]
CdSe/ZnCdS	28.6 ns	[4]
CdSe/$(CdS)_4/(ZnS)_2$	23.8 ns	[5]

Table S4. Time-resolved PL kinetics of CdSe@CdS@ZnSe upon the excitation of 400 nm. Fitting procedures are described by biexponential function analysis.

**Samples**	**Fitting parameters**				
	**τ_1 (ns)**	**A_1**	**τ_2 (ns)**	A_2	τ_{Avg} (ns)
**CdSe@CdS@ZnSe**	48.96	92.89	8.12	6243	11.5

References

1. Zi, J.; Zhong, Y.; Li, Z.; Wu, F.; Yang, W.; Lian, Z. Type-I CdSe/ZnS Heteronanoplatelets Exhibit Enhanced Photocatalytic Hydrogen Evolution by Interfacial Trap-Mediated Hole Transfer. *J. Phys. Chem. C* **2021**, *125*, 23945–23951.
2. Wang, X.; Qu, L.; Zhang, J.; Peng, X.; Xiao, M. Surface-Related Emission in Highly Luminescent CdSe Quantum Dots. *Nano Lett.* **2003**, *3*, 1103–1106.
3. Drijvers, E.; De Roo, J.; Geiregat, P.; Fehér, K.; Hens, Z.; Aubert, T. Revisited Wurtzite CdSe Synthesis: A Gateway for the Versatile Flash Synthesis of Multishell Quantum Dots and Rods. *Chem. Mater.* **2016**, *28*, 7311–7323.
4. Tao, C.-L.; Ma, J.; Wei, C.; Xu, D.; Xie, Z.; Jiang, Z.; Ge, F.; Zhang, H.; Xie, M.; Ye, Z.; et al. Scalable Synthesis of High-Quality Core/Shell Quantum Dots with Suppressed Blinking. *Adv. Opt. Mater.* **2023**, *11*, 2300533.
5. Wang, K.; Tao, Y.; Tang, Z.; Benetti, D.; Vidal, F.; Zhao, H.; Rosei, F.; Sun, X., Heterostructured core/gradient multi-shell quantum dots for high-performance and durable photoelectrochemical hydrogen generation. *Nano Energy* **2022**, *100*, 107524.
